# Supplementary material for: Adipose Triglyceride Lipase Deficiency Attenuates In Vitro Thrombus Formation without Affecting Platelet Activation and Bleeding In Vivo
Source: Cells. 2022 Mar 1;11(5):850. doi: 10.3390/cells11050850 (PMC8908992; doi:10.3390/cells11050850)
Supplement: Supplementary file 1 [file cells-11-00850-s001.zip › cells-1602937-supplementary.pdf]

Supplementary Figure S1

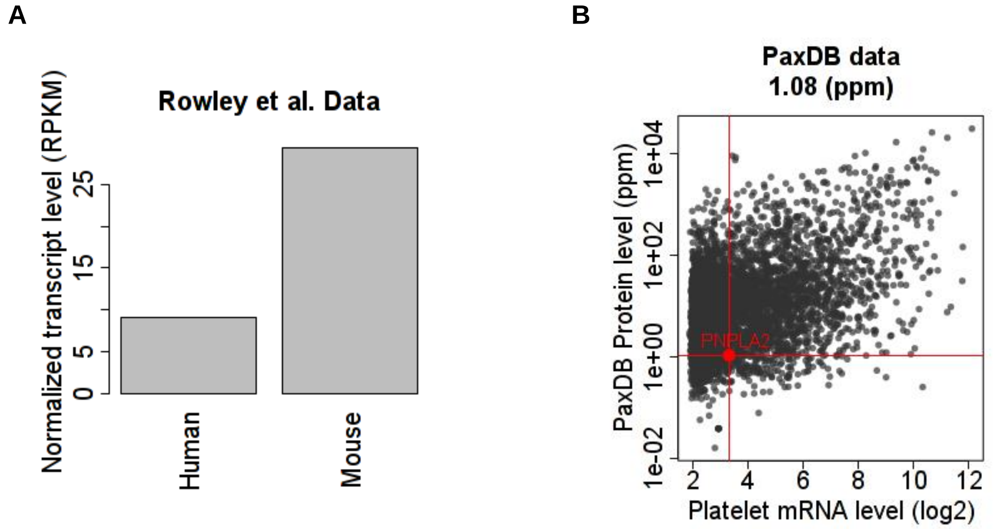

Figure S1. mRNA Expression of Atgl in human and mouse platelets. (**A**) Comparison between human and mouse mRNA expression of Atgl from pools of 2 human and 2 mouse samples determined by RNA sequencing (http://www.plateletomics.com/plateletomics/interactive-results?pAction=mRNA&pValue=PNPLA2,PNPLA2,7937485) [1]. (**B**) Correspondence between human platelet protein expression data and human platelet mRNA expression in 154 healthy subjects. Protein data is expressed in parts per million (ppm) from PaxDb: Protein Abundance Across Organisms (https://pax-db.org/) with a Spearman correlation coefficient of 0.30 and $p = 2.3e\text{-}99$ among the 4690 common genes assayed in both datasets [1].

Reference

1. Rowley, J.W.; Oler, A.J.; Tolley, N.D.; Hunter, B.N.; Low, E.N.; Nix, D.A.; Yost, C.C.; Zimmerman, G.A.; Weyrich, A.S. Genome-wide RNA-seq analysis of human and mouse platelet transcriptomes. *Blood* **2011**, *118*, e101-111, doi:10.1182/blood-2011-03-339705.

Supplementary Figure S2

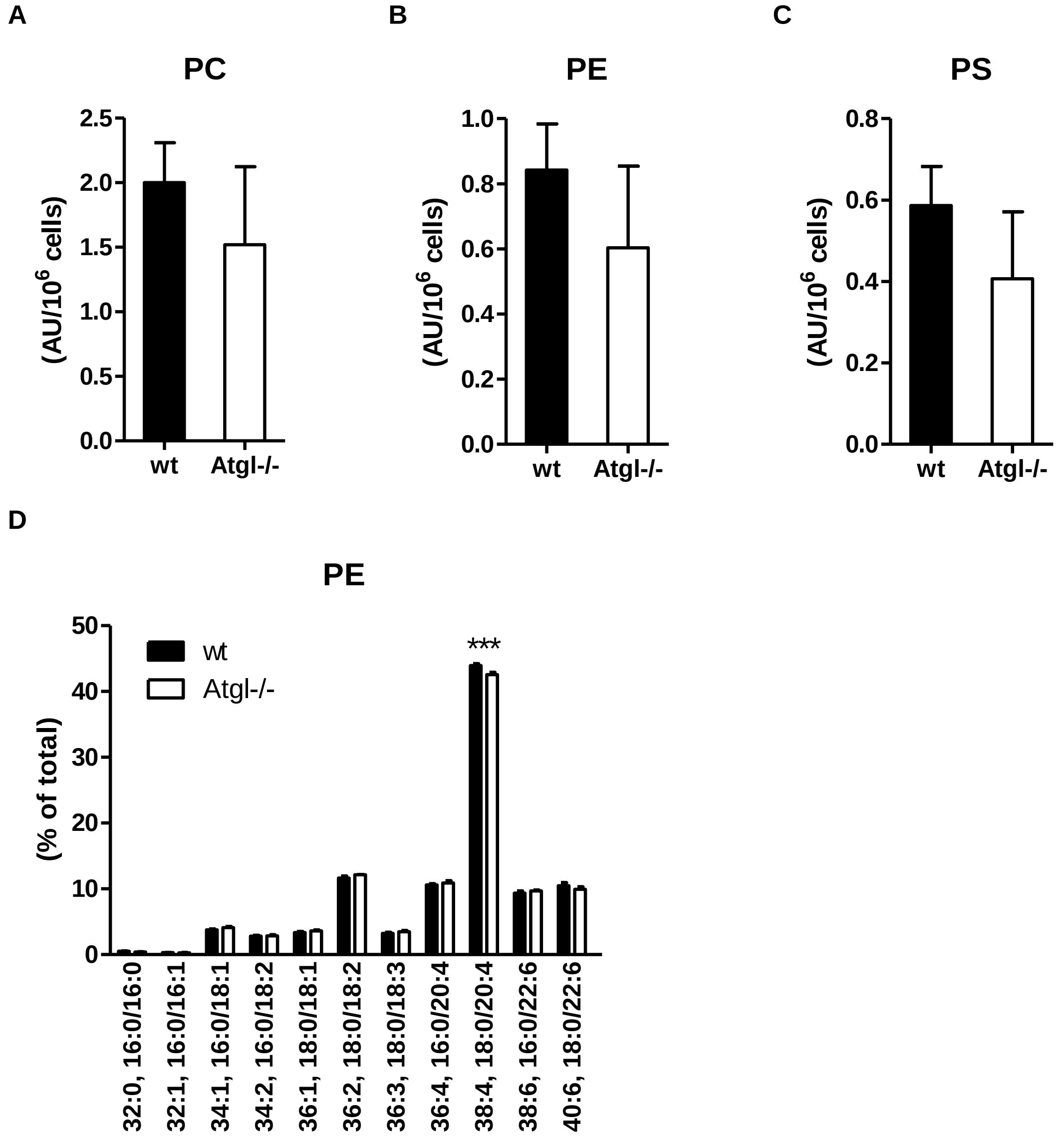

Figure S2. Unaltered phospholipid levels but altered concentrations of phosphatidylethanolamine species in Atgl-/- platelets. Platelet lipids were analyzed by UPLC-MS for (**A**) phosphatidylcholine (PC), (**B**) phosphatidylethanolamine (PE), and (**C**) phosphatidylserine (PS) concentrations as well as (**D**) PE species. Data are expressed as means + SEM (n=5). ***, $p < 0.001$.
